# Supplementary material for: Orientation of non-spherical protonated water clusters revealed by infrared absorption dichroism
Source: Nat Commun. 2018 Jan 22;9:311. doi: 10.1038/s41467-017-02669-9 (PMC5778031; doi:10.1038/s41467-017-02669-9)
Supplement: Supplementary file 1 — Supplementary Information [file 41467_2017_2669_MOESM1_ESM.pdf]

a narrow chain

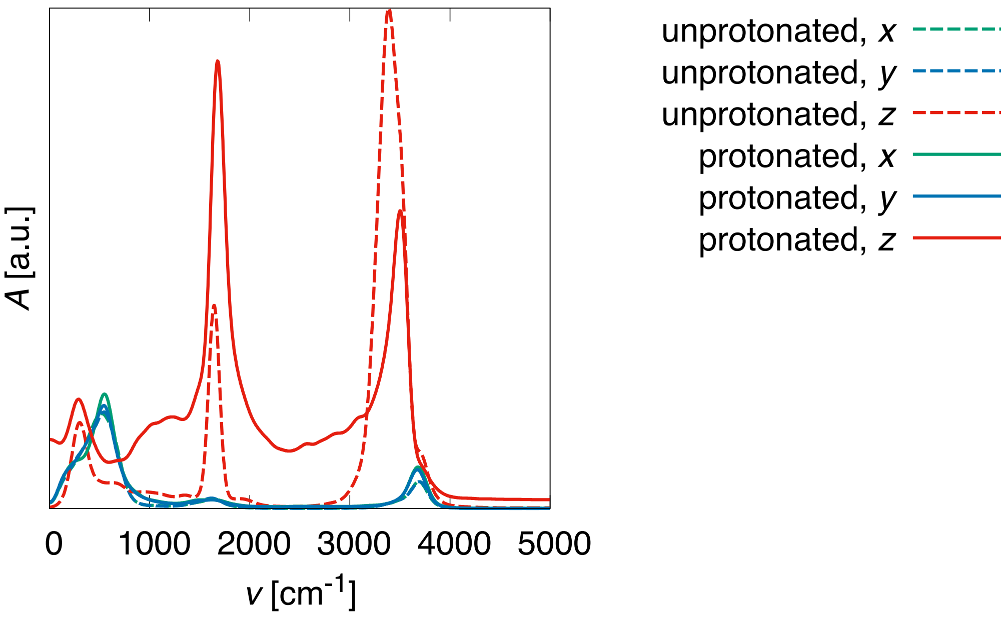

b wide chain

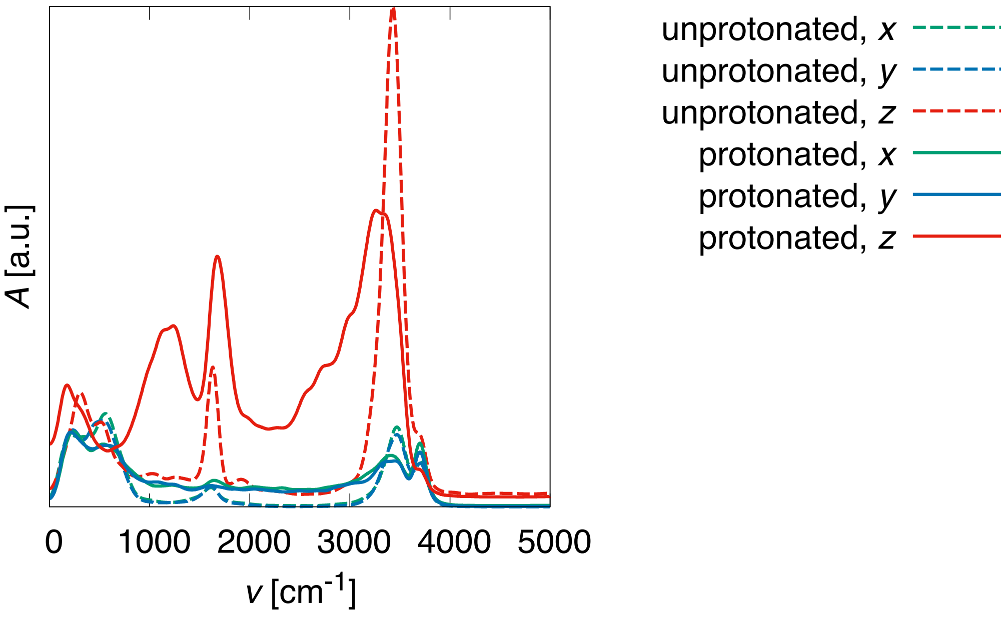

Supplementary Figure 1: Individual x, y and z contributions to the infrared spectra computed from ab initio trajectories for (**a**) a narrow chain and (**b**) a wide chain with (solid lines) and without (dashed lines) an excess proton. The differences between the x and y contributions are an estimate of the magnitude of statistical errors.

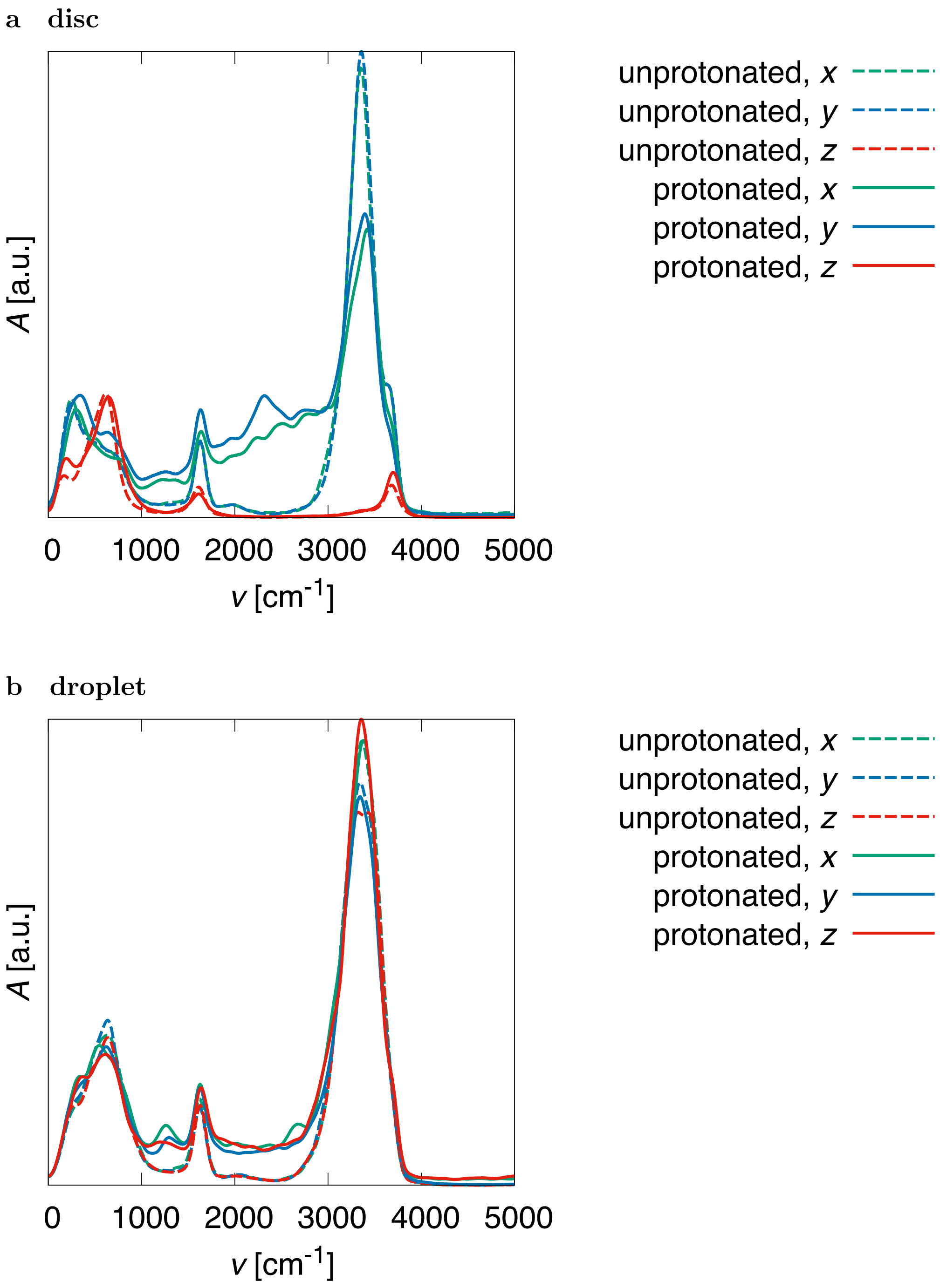

Supplementary Figure 2: Individual x, y and z contributions to the infrared spectra computed from ab initio trajectories for (**a**) a disc and (**b**) a droplet with (solid lines) and without (dashed lines) an excess proton. The differences between the x, y (and z for the droplet) contributions are an estimate of the magnitude of statistical errors.

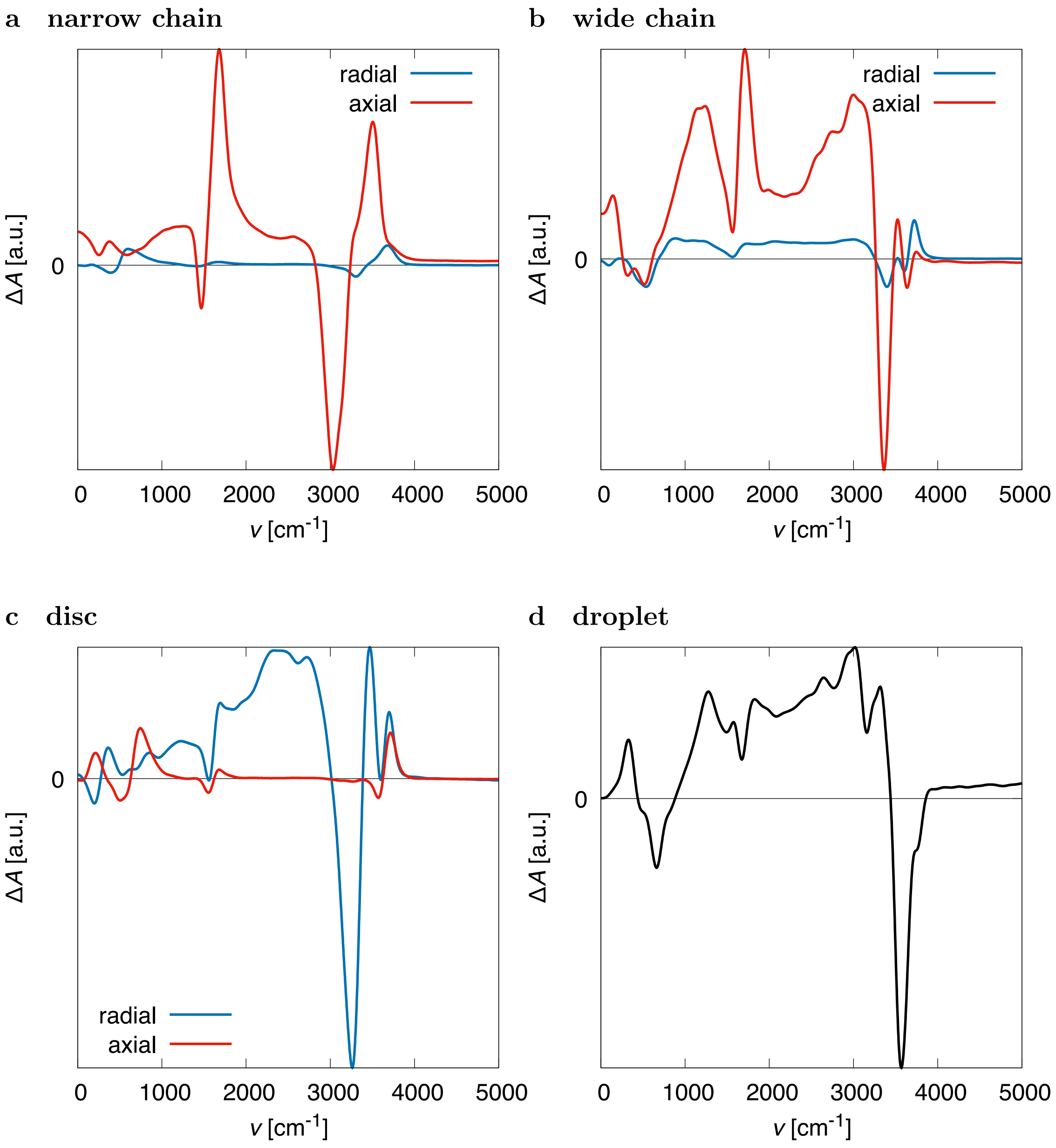

Supplementary Figure 3: Polarization-dependent protonated-unprotonated difference spectra computed from the ab ab initio trajectories for (**a**) a narrow chain, (**b**) a wide chain, (**c**) a disc and (**d**) a droplet.

Supplementary methods

We carried out the FTIR measurements with two samples, at two different hydration levels. The advantage of a controlled low hydration is that the photocycle of bR is slowed down[1,2]. As a consequence, the fraction of proteins that are trapped in a photostationary state is increased, leading to a higher signal intensity. As in this work we discuss difference bands, signal intensity is important to exclude baseline drifts and to improve the signal-to-noise ratio. The results shown in Fig. 2b in the main text have been measured with a sample 1 that is hydrated with a glycerol/water mixture of 8:2 wt/wt. From the absorption spectrum shown in Supplementary Fig. 6 we estimate the molar fraction of water and bR in the film, resulting in 750 ± 70 water molecules per protein monomer. As a control, we performed the same measurements with a more hydrated sample (4:6 wt/wt glycerol/water, sample 2), where we estimate a fraction of 1000 ± 100 water molecules per protein monomer. This sample shows a signal 3.8 times smaller in intensity compared to sample 1 (see Supplementary Fig. 4), but is almost indistinguishable in terms of band shapes and positions (see Supplementary Fig. 5), especially in the continuum band region.

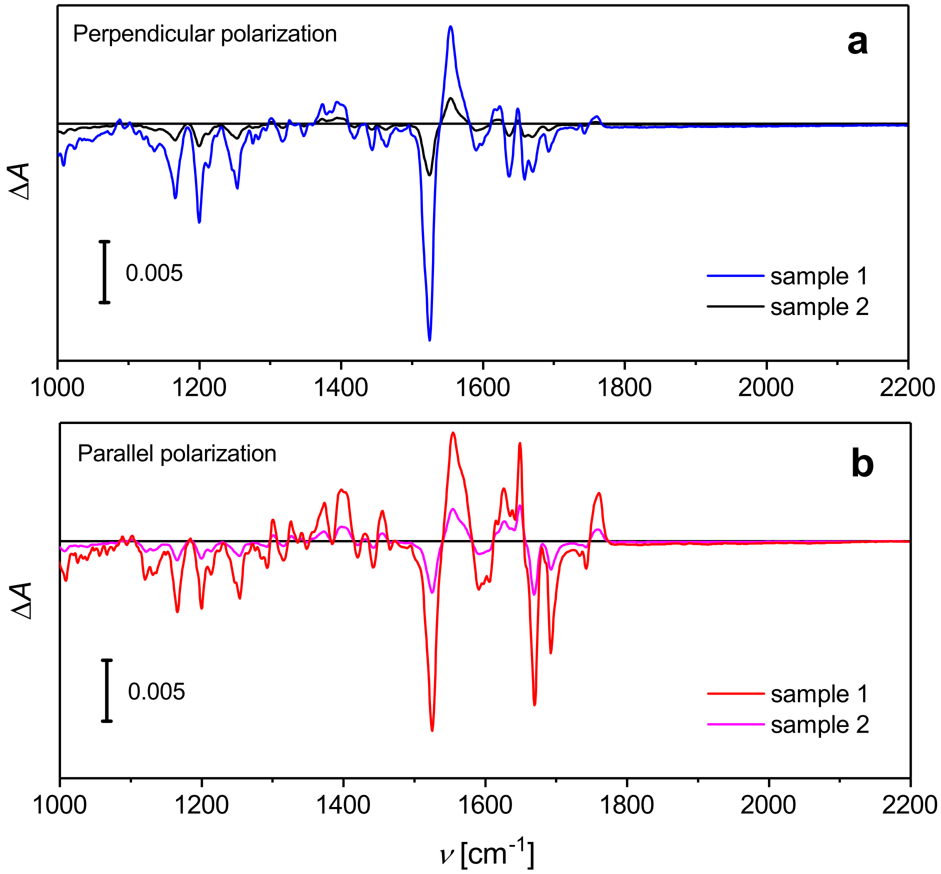

Supplementary Figure 4: Experimental light-dark FTIR-ATR difference absorption spectra of bR for the two different samples, with (**a**) perpendicular and (**b**) parallel polarized light (see Fig. 2a in the main text for the definition of the polarization directions). A higher hydration condition leads to a faster photocycle. As a consequence, a smaller fraction of bR molecules is accumulated in the M (and N) state under steady-state illumination. Thus, the light-dark IR difference spectra become less intense.

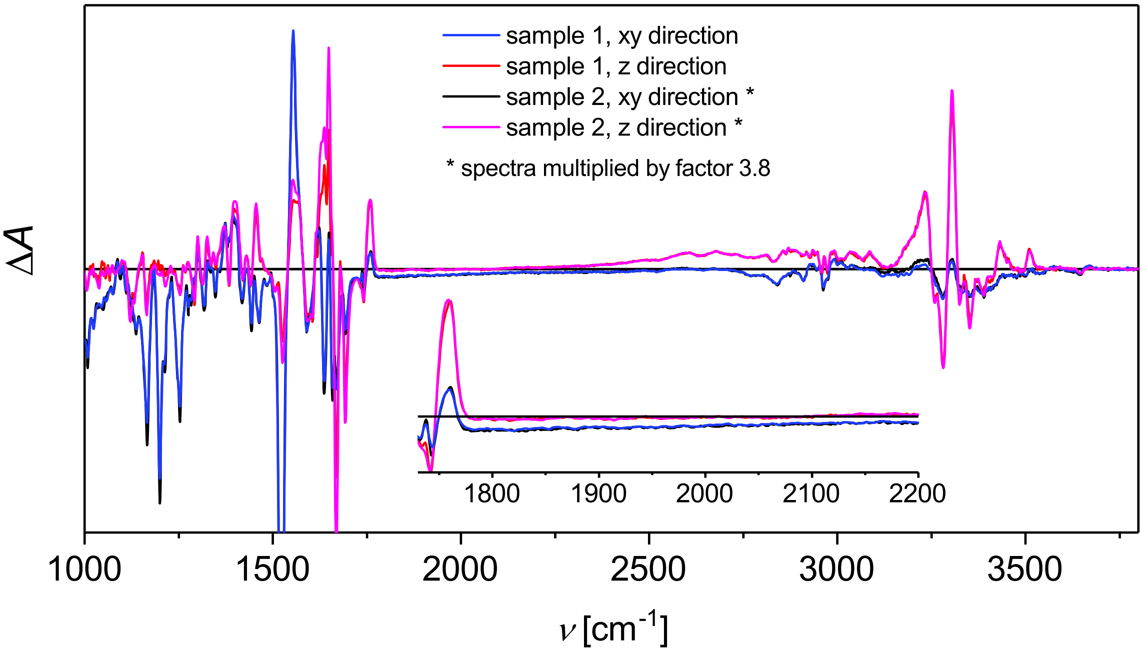

Supplementary Figure 5: Extracted experimental xy and z difference absorption spectra for the two differently hydrated samples; when scaled to each other (scaling factor 3.8) the respective signals significantly overlap over the whole range, and they are indistinguishable in the continuum band region (shown in the inset). This result confirms that a reduced hydration only increases the fraction of the photoaccumulated intermediates M (and N) under steady-state illumination conditions, without altering their spectra significantly.

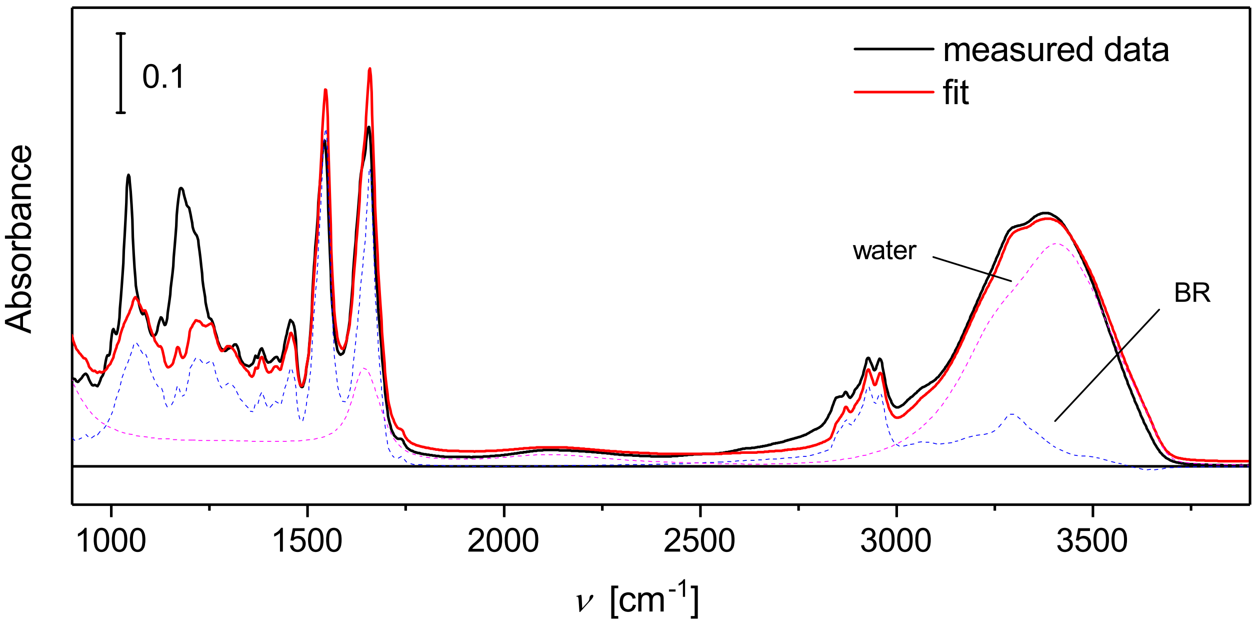

Supplementary Figure 6: Experimental infrared ATR absorption spectrum of sample 1. The molar fraction of water and bR molecules is estimated by fitting the absorption spectra measured with ATR and horizontally polarized light (black line) to the known extinction coefficient of liquid water[3] (pink line) and the estimated extinction coefficient of bR in the xy direction (blue line)[4]. We took into account that in ATR measurements the effective penetration decreases approximately linearly with the wavenumber. According to the fit, we estimate the presence of 750 ± 70 water molecules per protein monomer.

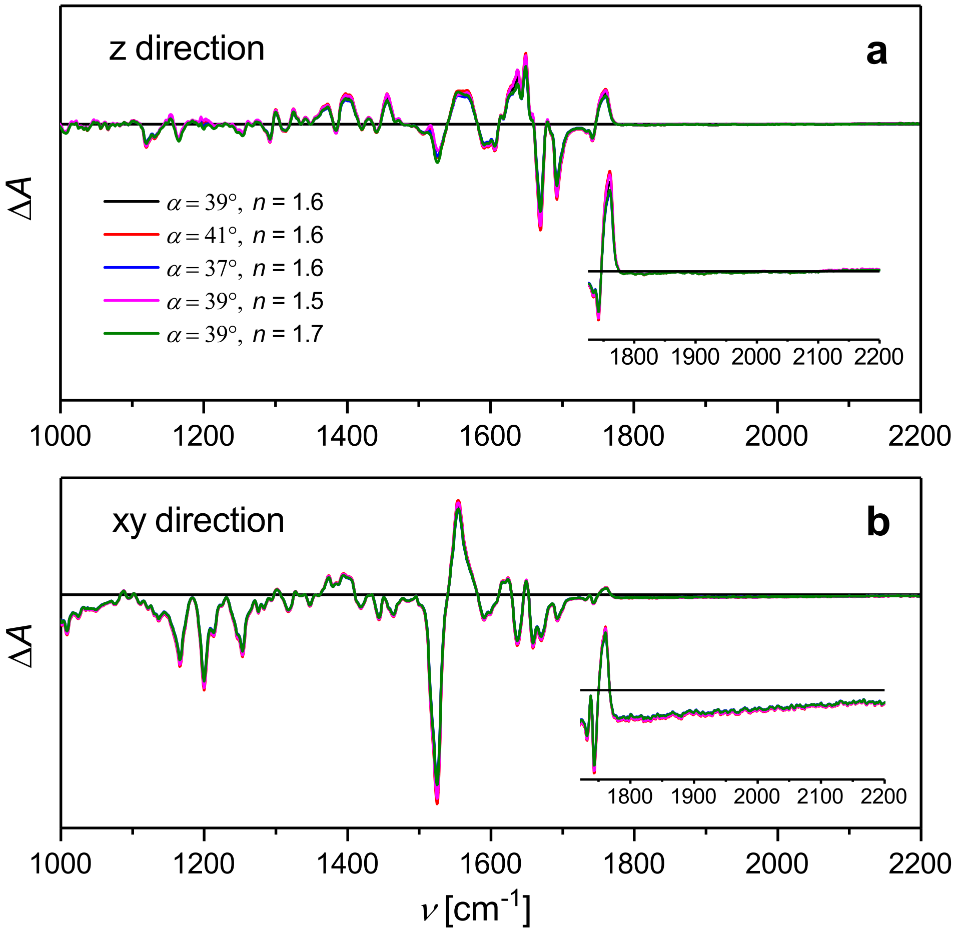

Supplementary Figure 7: Extracted experimental (**a**) z and (**b**) xy difference absorption spectra obtained using the thick-film weak-absorber approximation of Harrick[5] and assuming a range of different values for the refractive index n of the sample and of the incident angle α of the IR beam on the ATR surface. No significant differences can be observed.

Supplementary Note 1: Calculating IR spectrum contributions of the nuclear motion

The spectral contributions of nuclear motion neglecting electronic polarization effects (n) shown in Fig. 3 in the main text for the protonated narrow chain are calculated from the ab initio trajectories by assigning effective charges to the nuclei, but ignoring the electronic degrees of freedom. For protonated clusters, this is a crude approximation since the effective nuclei charges near the excess proton differ from the effective charges elsewhere. However, the total absorbance of unprotonated clusters is small in the frequency range of the continuum band between $2000\,\mathrm{cm}^{-1}$ and $3000\,\mathrm{cm}^{-1}$. This observation motivates our choice $q_\mathrm{H} = +e$ and $q_\mathrm{O} = -2e$. The total excess charge of the system is thus correct and localized near the excess proton. The contribution of neutral water molecules is overestimated, which explains why the vibrational peaks are too large compared to the $n{+}e$ calculation including electronic degrees of freedom.

Supplementary Note 2: Spectral projection of Zundel complex contributions

For the projection of the spectrum on the Zundel ensemble, we define an instantaneous projection operator $P_\mathrm{Z}^\mathrm{inst}(t)$ that selects all nuclei belonging to a Zundel complex, based on the asymmetry coordinate δ as discussed in Supplementary Note 3. Here we choose $|\delta| < \delta_\mathrm{thr} = 0.05\,\text{Å}$ to identify a Zundel complex. The nuclei belonging to a Zundel state are the central excess proton, the two neighboring oxygen nuclei O_1 and O_2 as well as the four additional protons coordinated to the oxygen nuclei O_1 and O_2. When no Zundel complex is found, we define $P_\mathrm{Z}^\mathrm{inst}(t) = \emptyset$. Clearly, this definition of $P_\mathrm{Z}^\mathrm{inst}(t)$ depends on the choice of δ_thr. Furthermore, the resulting trajectory pieces are in general too short for a proper spectroscopic analysis. Motivated by the fact that in the trajectories rapid oscillations of the excess proton between two neighboring water molecules are often observed, we define a retarded Zundel operator $P_\mathrm{Z}^\mathrm{ret}(t)$ by keeping the nuclei identified by $P_\mathrm{Z}^\mathrm{inst}(t)$ until a new Zundel state is found, i.e.

$$P_\mathrm{Z}^\mathrm{ret}(t) = P_\mathrm{Z}^\mathrm{inst}(\max\{t' \,|\, t' \leq t \text{ and } P_\mathrm{Z}^\mathrm{inst}(t') \neq \emptyset\}). \quad (1)$$

From the resulting set of trajectories after projection by $P_\mathrm{Z}^\mathrm{ret}(t)$, we calculate the center-of-charge trajectory for the retarded Zundel ensemble from the trajectories of the nuclei and the coordinated Wannier centers. Since the nuclei indices change due to the proton hopping process, these trajectories are not continuous. To account for this, we make the trajectory continuous by subtracting the corresponding offsets. We use these trajectories to compute the spectra of the Zundel state (included in Fig. 3 in the main text) for both the $n{+}e$ and the n ensemble. We remark that in a recent publication a similar method has been used to calculate infrared spectral signatures of protonated water clusters in bulk water[6].

Supplementary Note 3: Zundel occupation probabilities

We identify Zundel states based on the asymmetric stretch coordinate $\delta = d_{\mathrm{O_1H}} - d_{\mathrm{O_2H}}$, where $d_{\mathrm{O_1H}}$ and $d_{\mathrm{O_2H}}$ denote the distances between a proton and the two nearest oxygen nuclei O_1 and O_2[7], see Supplementary Fig. 8 for an illustration. We define a Zundel complex by the criterion $|\delta| < \delta_\mathrm{thr}$. In

Supplementary Fig. 9, the Zundel occupation probability calculated from the ab initio trajectories is given as a function of the threshold δ_{thr} for all four protonated water cluster geometries. Despite the fact that there is no canonical choice for δ_{thr}, the curves clearly show that the Zundel state is occupied significantly more often in the linear geometries than in discs and droplets. Note that we observe the lowest Zundel occupation number for the disc system, suggesting that the Eigen complex is stabilized in discs compared to bulk water.

For completeness, we show in Supplementary Fig. 10 the two-dimensional probability distributions $\rho(\delta, d_{\mathrm{O_1O_2}})$ of the asymmetric stretch δ and the oxygen distance $d_{\mathrm{O_1O_2}}$[7]. The plots confirm the above conclusion that the Zundel state, which corresponds to small $|\delta|$ and small $d_{\mathrm{O_1O_2}}$, is realized more frequently in chains than in discs and droplets.

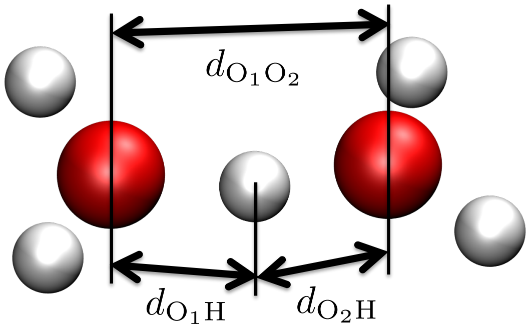

Supplementary Figure 8: Illustration of a Zundel cation and the asymmetric stretch coordinate $\delta = d_{\mathrm{O_1H}} - d_{\mathrm{O_2H}}$.

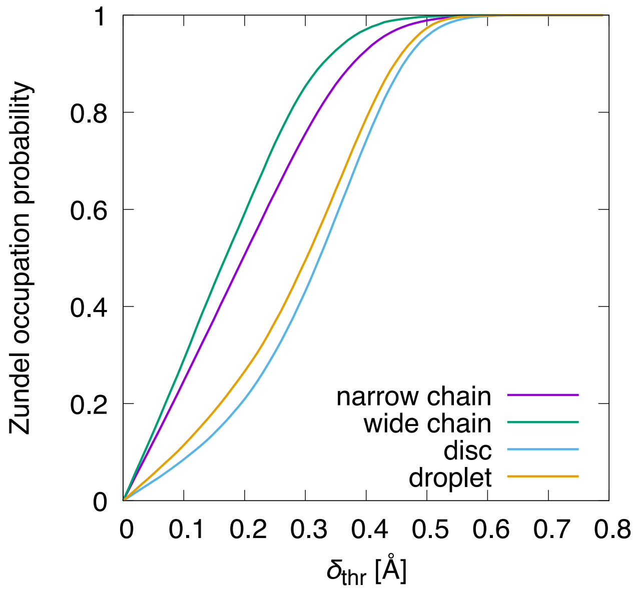

Supplementary Figure 9: Occupation probability of the Zundel state as a function of the asymmetric stretch threshold δ_{thr} for the different protonated water cluster geometries.

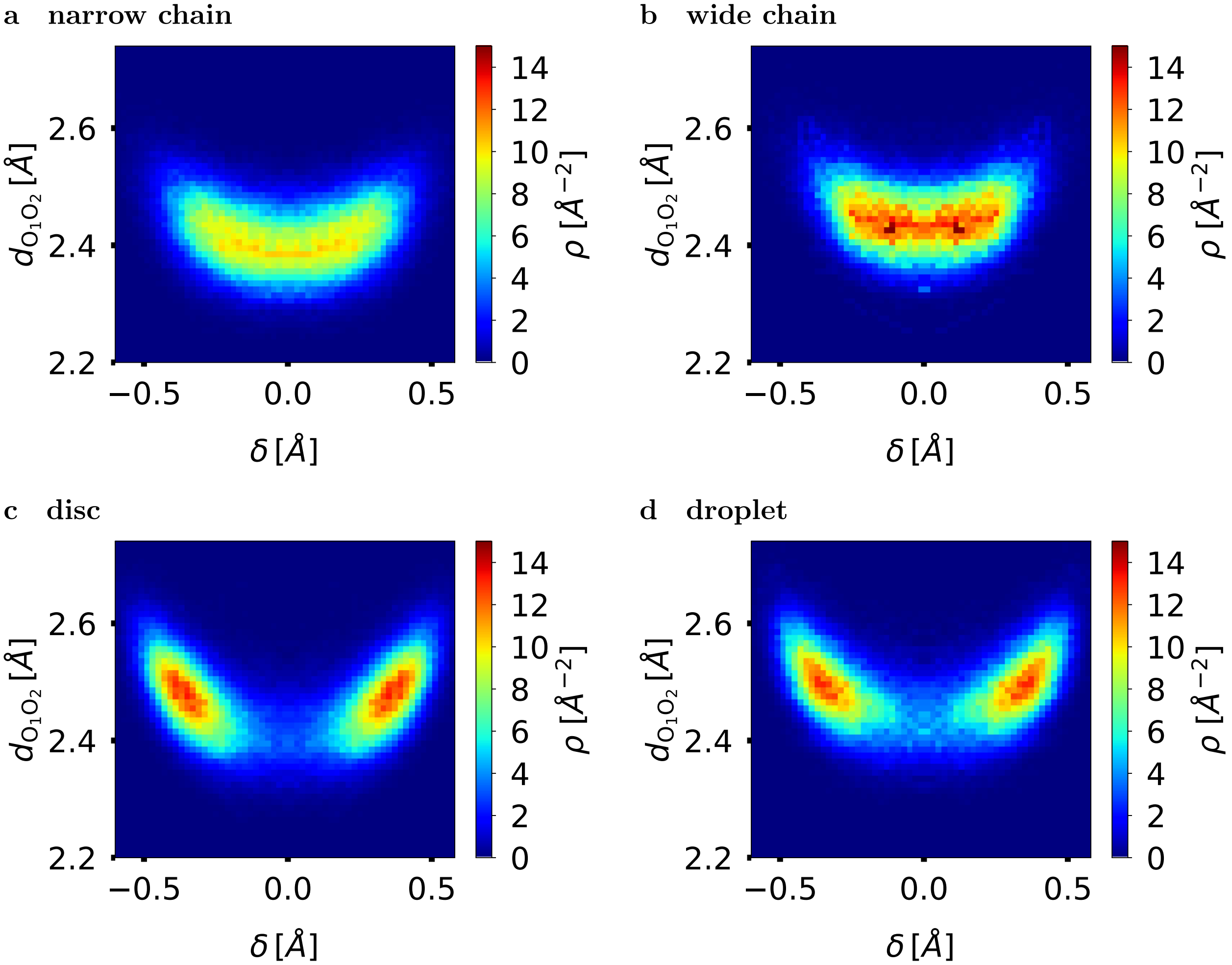

Supplementary Figure 10: The probabilty distribution $\rho(\delta, d_{O_1O_2})$ for the proton with the smallest asymmetric stretch with respect to its two nearest oxygen nuclei. Small $|\delta|$ and small $d_{O_1O_2}$ correspond to Zundel states.

Supplementary Note 4: Determination of the number of water molecules and confinement potential strengths for the disc and the droplet simulations

Disc. For the disc, the confinement potential strength is determined from two-dimensional classical water simulations using the GROMACS 4.6[8] simulation package and the SPC/E[9] water model. The oxygen atoms are constrained to a two-dimensional, periodic plane along the x and y direction (the plane has dimensions of $3.5\,\mathrm{nm} \times 3.5\,\mathrm{nm}$ and is embedded in a $3.5\,\mathrm{nm} \times 3.5\,\mathrm{nm} \times 4\,\mathrm{nm}$ box). We simulate an NPT ensemble for $5\,\mathrm{ns}$ using a v-rescale thermostat[10] at $T = 300\,\mathrm{K}$ and a Berendsen barostat[11] at $P = 1\,\mathrm{bar}$ coupled only to the x and y directions. In Supplementary Fig. 11a, we show the unnormalized radial distribution function $dN(r)/dr$ obtained from the last $1.5\,\mathrm{ns}$, where $N(r)$ denotes the number of oxygen atoms within radius r with respect to an oxygen atom. The peak structure implies that within $r_0 \approx 0.64\,\mathrm{nm}$, two circular shells of water around a central molecule

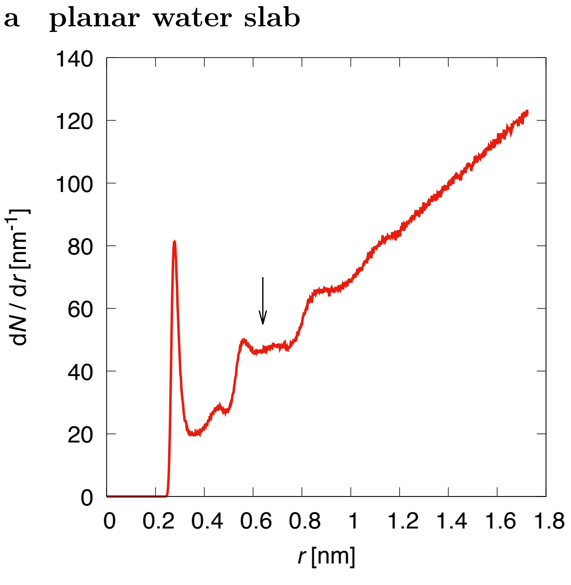

Supplementary Figure 11: Unnormalized radial distribution functions $dN(r)/dr$ for (**a**) a two-dimensional periodic SPC/E water plane and (**b**) three-dimensional bulk SPC/E water, where $N(r)$ denotes the number of oxygen atoms within radius r with respect to an oxygen atom. The radii r_0 are indicated by arrows.

are present. Integration of the data yields

$$\int_0^{r_0} dr \, \frac{dN(r)}{dr} \approx 15, \tag{2}$$

i.e. on average 15 oxygen atoms are found within r_0. The confinement potential strength $K_{xy} = 30\,\mathrm{kJ\,mol^{-1}\,nm^{-2}}$ for the ab initio simulations is chosen so that the 15 water molecules typically stay within the radius $r_0 \approx 0.64\,\mathrm{nm}$, implying that the planar water density in the ab initio simulation roughly agrees with the value from the classical simulation with periodic boundary conditions.

Droplet. For the droplet, we simulate a cubic water box of volume $(3\,\mathrm{nm})^3$, with isotropic pressure[11] and temperature[10] coupling. We show $dN(r)/dr$ in Supplementary Fig. 11b and find that two hydration shells of water are found within a radius $r_0 \approx 0.57\,\mathrm{nm}$, corresponding to 26 water molecules. To confine the droplet in the ab initio simulations, we choose a force constant of $K_r = 40\,\mathrm{kJ\,mol^{-1}\,nm^{-2}}$, which yields the same density as in the classical simulation of bulk water.

Supplementary references

1. Korenstein, R. & Hess, B. Hydration effects on the photocycle of bacteriorhodopsin in thin layers of purple membrane. *Nature* **270**, 184–186 (1977).

2. Thiedemann, G., Heberle, J. & Dencher, N. A. Bacteriorhodopsin pump activity at reduced humidity. In *Structures and Functions of Retinal Proteins Colloque INSERM/John Libbey Eurotext Ltd*, vol. 221, 217–220 (1992).

3. Bertie, J. E., Ahmed, M. K. & Eysel, H. H. Infrared intensities of liquids. 5. Optical and dielectric constants, integrated intensities, and dipole moment derivatives of H_2O and D_2O at 22°C. *J. Phys. Chem.* **93**, 2210–2218 (1989).

4. Lorenz-Fonfria, V. A., Saita, M., Lazarova, T., Schlesinger, R. & Heberle, J. pH-sensitive vibrational probe reveals a cytoplasmic protonated cluster in bacteriorhodopsin. *Proc. Natl. Acad. Sci. U. S. A.* **114**, E10909–E10918 (2017).

5. Goormaghtigh, E., Raussens, V. & Ruysschaert, J.-M. Attenuated total reflection infrared spectroscopy of proteins and lipids in biological membranes. *Biochim. Biophys. Acta, Bioenerg.* **1422**, 105–185 (1999).

6. Kulig, W. & Agmon, N. A 'clusters-in-liquid' method for calculating infrared spectra identifies the proton-transfer mode in acidic aqueous solutions. *Nat. Chem.* **5**, 29–35 (2013).

7. Marx, D. Proton Transfer 200 Years after von Grotthuss: Insights from Ab Initio Simulations. *ChemPhysChem* **7**, 1848–1870 (2006).

8. Hess, B., Kutzner, C., van der Spoel, D. & Lindahl, E. GROMACS 4: Algorithms for Highly Efficient, Load-Balanced, and Scalable Molecular Simulation. *J. Chem. Theory Comput.* **4**, 435–447 (2008).

9. Berendsen, H. J. C., Grigera, J. R. & Straatsma, T. P. The missing term in effective pair potentials. *J. Phys. Chem.* **91**, 6269–6271 (1987).

10. Bussi, G., Donadio, D. & Parrinello, M. Canonical sampling through velocity rescaling. *J. Chem. Phys.* **126**, 014101 (2007).

11. Berendsen, H. J. C., Postma, J. P. M., van Gunsteren, W. F., DiNola, A. & Haak, J. R. Molecular dynamics with coupling to an external bath. *J. Chem. Phys.* **81**, 3684–3690 (1984).
